# A Dynamic Dashboarding Application for Fleet Monitoring Using Semantic Web of Things Technologies

**DOI:** 10.3390/s20041152

**Published:** 2020-02-20

**Authors:** Sander Vanden Hautte, Pieter Moens, Joachim Van Herwegen, Dieter De Paepe, Bram Steenwinckel, Stijn Verstichel, Femke Ongenae, Sofie Van Hoecke

**Affiliations:** IDLab, Ghent University - imec, Technologiepark-Zwijnaarde 122, 9052 Gent, Belgium

**Keywords:** dynamic dashboards, Semantic Web of Things, Industry 4.0, fleet monitoring, SSN ontology, Web Thing Model

## Abstract

In industry, dashboards are often used to monitor fleets of assets, such as trains, machines or buildings. In such industrial fleets, the vast amount of sensors evolves continuously, new sensor data exchange protocols and data formats are introduced, new visualization types may need to be introduced and existing dashboard visualizations may need to be updated in terms of displayed sensors. These requirements motivate the development of dynamic dashboarding applications. These, as opposed to fixed-structure dashboard applications, allow users to create visualizations at will and do not have hard-coded sensor bindings. The state-of-the-art in dynamic dashboarding does not cope well with the frequent additions and removals of sensors that must be monitored—these changes must still be configured in the implementation or at runtime by a user. Also, the user is presented with an overload of sensors, aggregations and visualizations to select from, which may sometimes even lead to the creation of dashboard widgets that do not make sense. In this paper, we present a dynamic dashboard that overcomes these problems. Sensors, visualizations and aggregations can be discovered automatically, since they are provided as RESTful Web Things on a Web Thing Model compliant gateway. The gateway also provides semantic annotations of the Web Things, describing what their abilities are. A semantic reasoner can derive visualization suggestions, given the Thing annotations, logic rules and a custom dashboard ontology. The resulting dashboarding application automatically presents the available sensors, visualizations and aggregations that can be used, without requiring sensor configuration, and assists the user in building dashboards that make sense. This way, the user can concentrate on interpreting the sensor data and detecting and solving operational problems early.

## 1. Introduction: Evolution of Dashboard Architectures to Support for the Internet of Things

With the uprise of Internet of Things in industry, appliances and industrial machines are equipped with sensors for remote monitoring. The resulting amount of sensor data is overwhelming and hence difficult to interpret as-is. Therefore, dashboards are often used for aggregating the sensor data into visualizations that present a clear overview of a limited number of operational parameters (KPIs) in an instant.

Traditional dashboards, such as those in References [1,2,3], consist of a set of visualizations chosen in advance and are designed for a specific set of data sources; for example, a dashboard composed of gauges displaying the current temperatures in the five ovens in an industrial bakery. Such fixed-structure dashboards make the sensor data inspection easier, but they also restrain the user to the use cases of the dashboard that were agreed upon during its development phase.

Appliances or industrial machines may be part of a so-called fleet [4] of similar devices. Such fleets are often dynamic in nature—devices can be added to or removed from the fleet; sensors can be added to the devices, removed or replaced; and new types of sensors can be installed in the fleet. Such changes in the fleet must be registered in a dashboarding application, by modifying its code or its configuration, in order to keep the visualizations up to date. The introduction of new sensors in an industrial fleet may also require development of new aggregations and/or visualizations. As a result, traditional fixed-structure dashboarding applications require continuous investments in development and configuration effort such that they remain a practical monitoring tool for the evolving fleet. Changes to a dashboard require interventions by skilled people that know the internals of the fleet and the dashboarding software.

To overcome this issue, a next generation of dashboards was introduced, known as self-service dashboards or dynamic dashboards. They allow their end users to add, update and remove their personal visualizations in an intuitive way.

A good example of this approach is the Dynamic Dashboard Based on Activity and Reporting Traces (DDART) [5], a dashboarding platform for people that learn a skill by working on assigned challenges (project-based learning, PBL). With the dashboard, the participants can, for example, investigate the time spent per week on writing a project report. To do so, they can drag and drop (and further configure) scales (e.g., a datetime scale) and parameters (e.g., time spent per day) to the visualization’s axes, an aggregation (e.g., summing hours per day to hours per week) and an appropriate visualization. The basic process for composing widgets on a dashboard is similar in all dynamic dashboards, such as QlikView [6], Tableau [7] and Microsoft PowerBI [8].

The self-service visualization flexibility of these dashboarding platforms is delivered using an internally programmed myriad of if-then-else logic that expresses which visualizations fit with selected input data types. The flexibility comes at a cost—in some dashboarding platforms, such as DDART, the end user must have detailed knowledge about the data required for the visualization (e.g., know the function of each table in a database and indicate the data types of the columns to be visualized); in other platforms, an initial setup phase is required, in which such metadata is entered about all available data sources, after which end users can freely create visualizations.

Handcrafting visualizations however remains a tedious process that often draws the focus away from the problem at hand, since there often are a vast amount of data entities, filters, calculation functions and visualizations to select from. This may lead to choice overload for the end user, or to the creation of nonsensical or even non-functional visualizations. For example, on a dashboard monitoring air quality in offices, an end user might wish to visualize ${\mathrm{CO}}_{2}$ levels on a bar chart, not knowing that these ${\mathrm{CO}}_{2}$ levels have been saved with textual descriptions such as “high”, “medium” and “low”. A standard bar chart implementation will not be able to handle such input data.

To try and solve this issue, commercial dashboarding platforms adopted machine learning to recommend widgets automatically; for example, QlikSense [9] introduced natural language processing (NLP)—the platform creates the desired visualization based on the description of it, entered by the end user in his own words.

Such machine learning-based visualization recommenders require the creation of large supervised data sets (examples of user input and corresponding visualizations that were correct, as reported by the user) in order to train their skill. In the end, their results on new, never-seen user input is not guaranteed to always be exactly what the user wanted. Also, for visualization queries to be well understood, such as "average office temperature per floor", the setup phase in which the data must be annotated with its meaning, is still required, unless when queries are simple and data is stored in nicely structured database tables with clear column names. This approach meets its limits when all sensor observations are stored in different data formats on a message bus topic, or when observations must be queried on the fly from a set of web services.

Since a metadata annotation setup phase is required anyway, it is possible to save this metadata in a format that machines can understand without resorting to machine learning, using the Resource Description Framework (RDF) [10], a World Wide Web Consortium (W3C) standard for expressing knowledge in a machine-understandable format. The intelligent sensor-visualization matchmakings can then be suggested using semantic reasoners. After studying 23 dashboarding platforms, to the best of our knowledge, none of the commercial dashboards currently semantically annotate data sources, algorithms and visualizations with RDF. However, a lot of research has been done already on semantic annotation of data, some of which also focused on annotating and recommending visualizations. We will overview relevant research in this area in Section 3.

Another challenge to dynamic dashboarding software is the vast amount of sensors on the Internet of Thing to monitor in real-time. This requires extensive engineering in terms of scalability for data storage (e.g., long-term queryable backend storage of all sensor measurements) and software (e.g., efficient data aggregation). Some dashboarding platforms fail to adequately meet these requirements, which has led to open-source efforts that specialize in it, such as Mainflux [11].

Lastly, heterogeneousness of sensors deployed in industrial fleets also is a major challenge to dashboarding platforms. On the one hand, new types of sensors introduced in the fleet may require that a driver for yet another communication protocol and data exchange format must be added in the dashboarding software. Some dashboarding platforms, such as IBM Cognos [12], try to automate data format handling for web services using web service description languages, such as OpenAPI (formerly Swagger) [13] or JSONSchema [14] for REST services [15], and WSDL [16] for SOAP services [17]. On the other hand, all additions and removals of sensors and assets in the industrial fleet must be registered in the dashboarding platform, either manually on the user interface, programmatically by integration with an external, company-owned asset management system or by publishing changes to an API, for example, the ThingsBoard [18] REST API. It is, however, clear that, for any of these fleet management solutions, a lot of human effort is still required. Therefore, automatically discovering the presence of devices, such as sensors, and potentially even metadata about the assets they monitor, will evidently be of great added value. This subject has been covered in many research studies. Section 2 will discuss this in more detail.

In conclusion, a number of challenges for dynamic dashboards remain unsolved. Our literature overview showed that semantic annotation of sensors, data processing functionality and visualizations, combined with matchmaking rules, allow a semantic reasoner to suggest visualizations. We also argued that the annotation of expert knowledge may be more promising than using machine learning-based recommendations. In this paper, we therefore build a dynamic dashboarding approach using semantic Web of Things technologies. 

We define the following goals to be tackled in this paper:

1. Build a functional dynamic dashboarding application
  (a) Enable continuous monitoring of an industrial fleet
  (b) Enable interactive creation of visualizations
2. Reduce human effort with automated industrial asset management
  (a) Automate sensor discovery
  (b) Automate asset discovery
3. Reduce human effort with visualization suggestion, using semantic reasoning
  (a) Semantically annotate sensors, data processing functionality and visualizations
    - Annotate data types of sensor observations
    - Annotate functionality of visualizations and aggregations
    - Do not require more effort and technical expertise for metadata annotation
  (b) Suggest visualizations for (aggregated) sensor data, using semantic reasoning
    - Avoid visualization choice overload and creation of nonsensical and non-functional widgets
    - Keep the required time for reasoning acceptable for user experience (<1 second).
    - Do not require technical expertise or detailed knowledge about the fleet, sensors, data processing services and visualizations.

Some first steps have been taken to deliver dynamic dashboards with Semantic Web of Things technologies, as discussed in this section (and in more detail in Section 3), but to the best of our knowledge, we are the first to combine dynamic dashboarding, sensor-visualization suggestion using semantic reasoning and automated sensor discovery.

**Figure 1:**
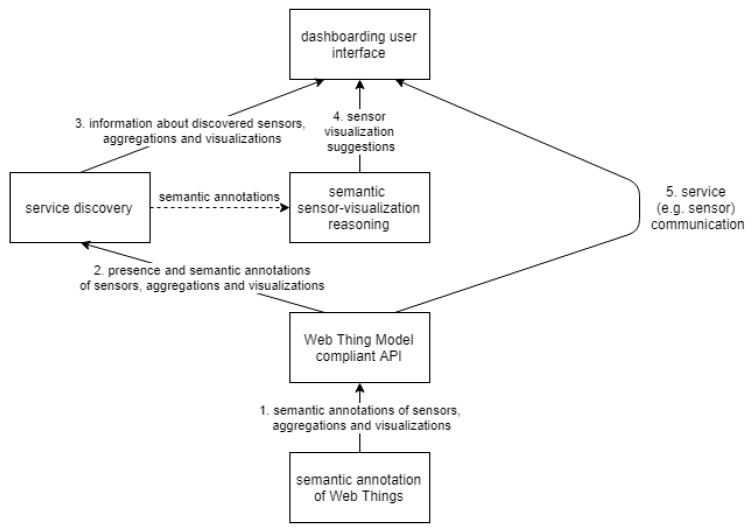
High level overview of actions involved in our approach for dynamic dashboarding. Note that arrows indicate flow of data, meaning that beforehand, a request for that data was made in the direction opposite to that of the arrow.

Figure 1 explains our approach on a high level, in five steps. First, a component is able to fetch or generate semantic annotations of the available Web Things, such as sensors, aggregations and visualizations. Then, a component discovers the presence and semantic annotations of these Web Things. This is achieved by crawling (walking over linked URLs) a Web Thing Model compliant API. This service discovery component is then able to send the dashboarding user interface, upon request, the list of discovered Web Things. With this, the user interface can present the sensors and aggregations that can be selected while building a new widget to be added to a dashboard. Once a user selected one or more sensors to visualize, and possibly an aggregation function to apply, a semantic reasoning component suggests appropriate visualizations for the selected sensor(s) and aggregation, given their semantic annotations, which were discovered earlier. Then, once the user selected one of the suggested visualization options, a widget will be created on the dashboard for the chosen visualization. Finally, the dashboard interface will start communicating with the Web Thing Model compliant API—it will fetch the visualization’s layout and code, load that into the created widget and start to continuously fetch the latest sensor observations, possibly process it using the aggregation service and eventually add the resulting data points to the visualization.

We note that one of our research goals is to evaluate the dynamic dashboard on an industrial fleet monitoring use case. We had the chance to do this on data produced by sensors on a train bogie, delivered by one of the partners within the imec.icon DyVerSIFy project.

In the remainder of this paper, we first discuss the three main drivers of our solution in detail—Web Thing discovery in Section 2, semantic annotation of Web Things in Section 3 and semantic reasoning about appropriate sensor visualizations in Section 4. Combining these sections, we present the conceptual architecture of our dynamic dashboarding software in Section 5. We present and discuss our results in Section 6 and also list some future work. Section 7 summarizes the added value of the presented solution.

## 2. Discovering Sensors and Services and Agreeing on Data Exchange Protocols

Dynamic dashboarding applications for real-time monitoring of fleets of industrial assets must discover a vast amount of sensors at run-time and integrate with a wide range of sensor communication protocols and data formats. In this section, we will discuss these challenges and work towards a solution.

The Industry 4.0 interconnection principle [19] dictates the industry to interconnect sensors, appliances and industrial machines over the Internet of Things (IoT) and encourages to reduce the number of sensor intercommunication protocols (e.g., MQTT, CoAP) by only using standard Web protocols (e.g., HTTP over TCP/IP) and providing RESTful [15] web APIs as a uniform interface [20,21,22]. Such web-enabled sensors are often called Web Things, and the web of interconnected Web Things is then called the Web of Things. The encapsulation of a sensor in a RESTful web API means that, for example, a simple HTTP GET request suffices to retrieve one or more of the sensor’s observations. This reduces the heterogeneity of sensor protocols that an integrator application, such as a dashboard, is faced with.

Encapsulating sensors as RESTful web APIs does not solve the lack of widely accepted service description languages, which is a frequently recurring issue in emerging IoT applications. When discovering the sensors, either encapsulated as RESTful web API or not, it is unclear what API endpoints exist (e.g., /data or /observations/latest), which requests must be sent to them, what the response will be and what the format of the content should be. If this information is documented, it is only documented for humans, therefore requiring human effort to integrate the API with a dashboard. Moreover, the so-called bootstrap problem [20,22,23] remains as well—for an application to retrieve the service definition and to communicate with the service, it must first be told or find out what sensor web APIs are available and what their address is (e.g., http://company.com/sensors/1).

These issues are particularly dramatic in the search for a dynamic dashboarding platform.

### 2.1. Sensor Discovery

Discovering available sensors is key in avoiding that sensor addresses must be configured one by one, by the dashboard user at run time, or by the developer at design time. In a local network, the bootstrap (discovery) problem can be solved using network discovery methods like DNS-SD, mDNS, UPnP, or protocol suites like DLNA or zeroconf. For these methods, devices added to the network generally obtain their IP address automatically from a DHCP server and broadcast it to other devices present on the local network using one of these announcement protocols.

However, these local network discovery methods don’t scale up to the Internet [23], nor do they work for dashboards that are deployed outside the network in which the fleet monitoring sensors are deployed (for example, the dashboarding software may run in the cloud but monitor a sensor network in a remote factory). One approach could be to securely expose the DHCP table [23,24] to the dashboard, however, for companies that maintain vast sensor networks, this means the software of many DHCP servers must be modified. Building a public search engine or private software component that crawls the Web for linked sensor APIs is also an option, but this will only work for RESTful sensor APIs that link to other APIs with REST’s building block HATEOAS [23]. So the most pragmatic solution is to just manually register a “root” address or address inside each sensor network in the dynamic dashboarding software. Then, upon HTTP GET request to that IP address and a predetermined TCP port, a discovery service responds with a list of the available services by using a local network discovery method.

### 2.2. The Web Thing Model

This Internet-scale approach, so far, does not inform about the vast amount of heterogeneous communication protocols and data formats used by the discovered Web Things. To avoid the need to implement a plethora of such definition languages, the Web Thing Model [22] was proposed, which is a W3C Submission formulating a contract that both RESTful sensor APIs and software clients can agree upon in order to make sensor services discoverable and to exchange data over the TCP, IP and HTTP protocols and with a agreed-upon JSON data format.

The Web Thing Model imposes three different integration patterns, namely direct connectivity, gateway-based connectivity, and cloud-based connectivity. Regardless of the chosen connectivity pattern, a set of RESTful API routes needs to be provided, each with their own goal. Table 1 gives an overview of these Web API routes. A sensor API that complies with the Web Thing Model contract is also called an Extended Web Thing.

As Table 1 shows, the API "contract" specifies web resources to find the sensors (Things) and sensor properties that are available. It also imposes a JSON data model to convey information about the sensor properties.

Note that a Web Thing API can expose sensor properties directly on the API’s root ({wt}/properties) when the direct connectivity integration pattern is applied (the Web Thing API represents a single sensor), or expose sensor properties on a Web Thing resource ({wt}/things/{thingId}/properties) when the other integration patterns are applied (the API serves as a proxy for one or more remote sensors).

Listing 1 shows a shortened JSON response, retrieved from the {wt}/things resource (http://localhost/web-thing-gateway/ in this particular case), that announces the availability of three Web Things on the Web Thing Model API—a latitude sensor, a concatenation service and a map visualization.

**Listing 1.** Shortened JSON response that a Web Things API provides on the http://localhost/web-thing-gateway/things resource, for three available Web Things—a sensor, aggregation and visualization.

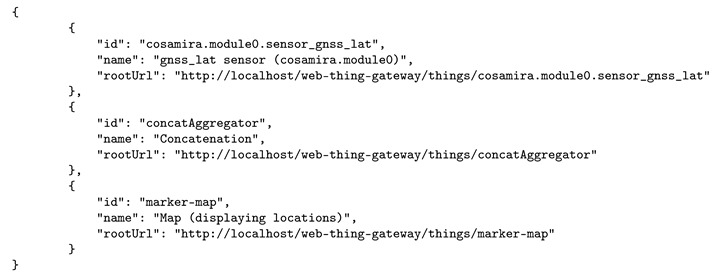



The standardized web resources on a Web Thing API allow software, such as dynamic dashboards, to construct a list of the provided sensors and the properties they observe, by crawling the available web resources on the API.

Annotating Web Things semantically, describing what their purpose is, allows a dynamic dashboarding application to also understand how to combine multiple Web Things. The Web Thing Model defines semantic extensions for this purpose, however proposes that the available web resources should have a HTTP Link header, referring to a JSON-LD context. We argue that content negotiation on the Web Thing’s URL also allows for semantically annotating the Web Thing, and thus provide a separate annotation in Turtle, using standard and custom ontologies, as will be described in Section 3. We semantically annotate things and properties. A crawler can then fetch these annotations.

As will also be discussed in Section 3, in the annotations of sensors, we also refer to the system that a sensor is part of, in terms of an industrial system hierarchy; for example an engine oil temperature sensor may be deployed in the engine of the left wing of an air plane. We assume that a web resource will also be provided for these systems, hosted by the Web Thing Model compliant Web API and semantically annotated, for example on a custom route {wt}/systems/1. This URL can then be linked to from within the semantic annotation of the sensor.

### 2.3. Discovering Visualisations

In the dynamic dashboarding application, visualization components are also web resources provided under /things by a Web Thing Model compliant web API. However, no properties are made available through the Web Thing Model API for these visualization Web Things, since they do not observe anything and therefore do not generate data. Additionally, we added content negotiation on the visualization web resources, by using the proper Accept header during the request, to support retrieving (i) the JavaScript file to inject into HTML-based dashboarding applications, (ii) the visualization’s layout (CSS) file, and (iii) the semantic annotation of the component.

### 2.4. Advantages and Disadvantages

In summary, the main advantage with the Web Thing Model is that it facilitates dynamic dashboards with, on one hand, automatic sensor and semantic annotation discovery, and, on the other hand, unifying the dashboard’s access to Web Things such as sensors with a single protocol and data format.

Because of the introduction of the Web Thing Model, the responsibility of configuring the presence of a new Web Thing within a known network, annotating its data types and implementing protocol bindings and data format mappings is moved from within the dashboarding software to the Web Thing API.

This has the advantage that the end user of the dashboarding software must not know the technical details about the sensors in the fleet and must not be a semantics expert in order to visualize sensor data.

A drawback is that Web Thing registration, semantic annotation, protocol driver implementation and Web Thing Model API implementation must still be performed in the Web Thing API by a technical expert such that a dynamic dashboard can discover the Web Things. However, the Web Thing Model API is a light-weight specification and therefore does not require a lot of implementation effort. Communication protocol drivers are already implemented in dashboarding software, they can be copied to the Web Thing API. Web Thing registration on the Web Thing gateway can be automated using the local network discovery methods discussed in this section. Semantic annotation effort can also be reduced, see Section 3 and Section 6.

Section 6 will discuss these findings with regard to the goals set in Section 1.

## 3. Semantic Annotation of the Available Web Things

To enable dynamic dashboarding applications to visualize sensors at will, the semantic reasoning needs knowledge about the abilities of the selected sensors and aggregations, and the available visualizations. In the following subsections, we discuss how sensors, visualizations and aggregations can be semantically annotated.

### 3.1. Related Work—Sensor Annotation and Visualization Suggestion in Dashboarding Research

A number of research papers about semantic sensor annotation and visualization, specifically for dashboarding, exist.

In Reference [25], the EnMonitor application displays near real-time measurements to citizens about environmental conditions. This dynamic dashboarding application continuously executes SPARQL queries to a sensor data store and visualizes the found observations. Sensor data is semantically annotated—the type of data observed by sensors is expressed using concepts from the FIESTA-IoT Ontology [26] and M3-Lite Taxonomy [27]. Visualizations are not annotated however, and as a result the presented dashboard still has a fixed structure.

In Reference [28], smart city data is converted to knowledge graphs, such that indicators can be discovered to compare cities. The Semantic BI Generator was proposed, an application that enables users to compose visualizations of the discovered city indicators. The city indicators are expressed in the KG using the ISO 37120 Indicator Definitions Ontology, a standard defining 100 indicators across 17 themes such as economy and education, but not the easiest ontology for creating visualizations from generated facts; and the developed QoE Indicators ontology, aimed at publishing city indicator values using concepts that allow for easier coupling with indicator visualization. These concepts include dimensions (e.g., length, width, depth) and measures (e.g., the length of a specific car is 3 m). The authors clearly state that interesting future work with their semantified indicators comprises inferring the best suited visualization types.

In Reference [29] both the data and the visualization services are semantically annotated. A user interface allows for selecting data to visualize. The user input is transformed to a SPARQL query, which is executed against the SPARQL endpoint available on the knowledge graph, generated from a relational database. The variables in the query’s result set are annotated with their data types, using the developed Label Ontology. Visualizations are represented using concepts from the developed Chart Ontology. Most importantly, a code template for constructing the visualization on the user interface is added in the semantic annotation. All facts about the SPARQL query result and visualizations are stored in the Instances Ontology and a semantic reasoner infers, using SWRL sensor-visualization matchmaking rules, which visualizations are appropriate for a given SPARQL result set. Finally, the user interface renders the layout of the first suggested visualization by inserting the data from the result set into the visualization’s code template. An end user may switch the suggested visualization with any other visualization that was deemed appropriate for the given SPARQL result set. The entire approach is a great foundation for any semantics-powered dynamic dashboarding software. However, its ontologies cannot be found and are coupled to the context of SPARQL result sets in particular. This also means that any data source should be wrapped with a data-to-RDF translation tool and a SPARQL endpoint, which is particularly a burden in case the status of sensors in large fleets of industrial machines must be visualized. Continuously monitoring such assets also requires that the SPARQL query is repeatedly run, which may pose scalability problems on the knowledge base infrastructure, and inserting new data points into the widgets will be more efficient than regenerating the widget entirely every time the query is executed.

### 3.2. Sensor Annotation

Vocabularies or ontologies can be used to semantically describe things. To annotate the available sensors, the Semantic Sensor Network (SSN) ontology [30,31] is used as it is the W3C-recommended and de facto standard ontology for modeling sensors, observed properties and observations. The SSN ontology is built on top of another ontology, namely the SOSA (Sensor, Observation, Sample, and Actuator) ontology [32].

Sensors can observe either quantitative (measurable, such as car speed or flow rate of a fluid through a pipe) or qualitative (not measurable, expressed with a “label”, such as an engine state, active or locked, open or closed). To capture the vast amount of specific sensor and property types, a number of vocabularies exist, such as the Quantities, Units, Dimensions and Types ontology [33], the Ontology of Units and Measurements (version 2, abbreviated as OM-2) [34] and the The Machine-to-Machine Measurement (M3) Lite Ontology (M3-lite) [27]. However, none of these also model specific qualitative properties, such as a machine status. Contrary, the Observable Property (OP) ontology [35] does model the quantitative/qualitative property distinction, but apart from that, it is a fairly limited-size ontology and does not contain any further interesting concepts for our dashboarding domain.

As we need this quantitative/qualitative distinction to find appropriate visualizations, a custom ontology was designed (part of our custom dashboarding ontology; for more detail, see Section 3.3). This way, we can annotate available sensor properties with their generic and specific type available using the dashb:QuantitativeProperty/dashb:QualitativeProperty distinction and the dashb:produces predicate from our custom ontology. The dashb:produces predicate links a property observed by a sensor with the specific metric (property type) it produces. These metrics are documented as reusable concepts in a custom metrics ontology, also using concepts from our custom ontology and the OM-2 ontology. Listing 2 shows the semantic annotations of a subset of the available sensors, more specifically those required for demonstrating the selected reasoning use cases.

Some concepts from the metrics ontology were used. Listing 3 displays the semantic knowledge available about the latitude and longitude metrics that were produced by the sensors in Listing 2. Note that we are not applying om:Temperature, om:hasUnit, dashb:category and dashb:dataType on the sosa:ObservableProperty instances directly, but use dashb:Metric instead, thereby avoiding that the dashboarding ontology and dashboarding metrics ontology is limited to only real-time monitoring (dashboarding) of sensor data.

**Listing 2.** Semantic annotation of two sensors, observing respectively the latitude and longitude of a position of a train.

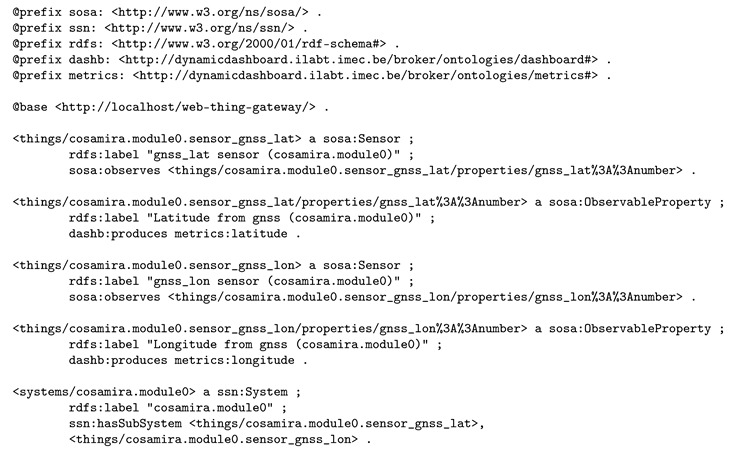



**Listing 3.** Semantic knowledge about the latitude and longitude metrics that were produced that were produced by the sensors introduced in Listing 2.

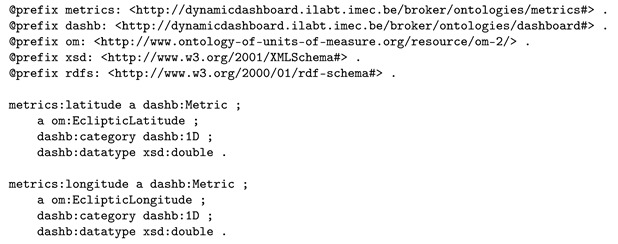



#### 3.3. Visualization Annotation

To enable the dashboard to suggest fitting visualizations for sensor properties and aggregations, the visualizations and their capabilities also need to be semantically described.

Existing ontologies for data visualization are either very limited on the number of visualizations they contain (e.g., the FaBiO ontology [36], which only contains a Gantt chart), or lack any properties for the visualisation type concepts (e.g., the Semanticscience Integrated Ontology (SIO) [37]). For the Visual Analytics vocabulary [38], we were unable to retrieve the vocabulary file or related software project.

**Figure 2:**
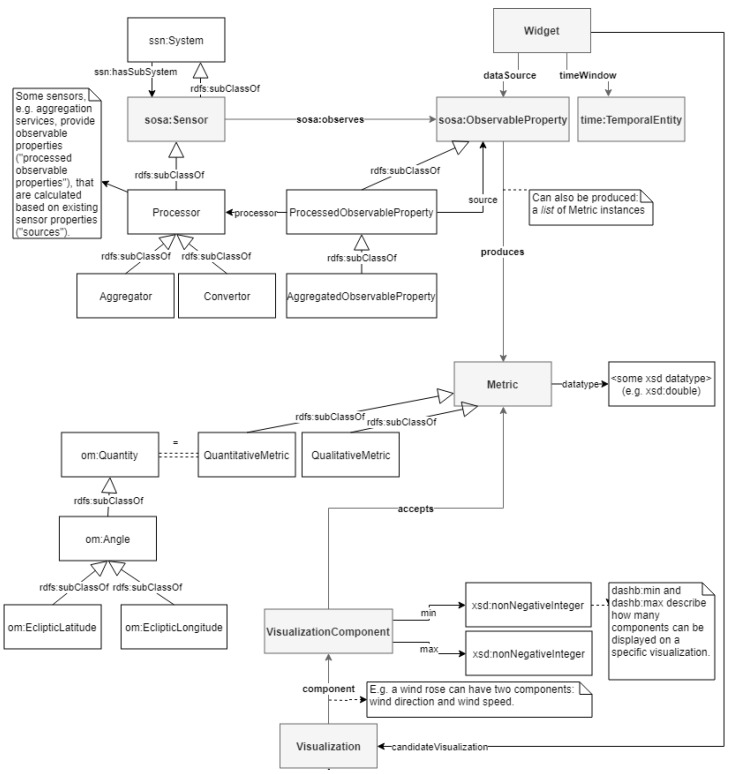
Classes and their relationships for annotating visualizers semantically, as designed in the dashboarding ontology.

As a result, and because more custom concepts were required to start reasoning about sensor-visualization suggestions, we created our own dashboarding ontology that allows to state that, for example, a column chart can visualize a maximum of 10 observations of quantitative sensor properties (observations of type xsd:double) per visualization update, whereas some map implementation only accepts one pair of latitude and longitude per visualization update. The quantitative resp. qualitative sensor property types are also integrated within this ontology. Figure 2 gives an overview of the most important classes.

The dashboarding ontology is open-source and is available on http://purl.org/dynamic-dashboard.

Implementations of visualizations must be semantically annotated in order for the dynamic dashboarding software to understand if they can match with sensors selected by the user. These annotations are made using concepts from the dashboarding ontology. Two examples are presented in Listing 4. They are for visualizations that respectively show positions and travelled routes on a map. Note that http://purl.org/dynamic-dashboard also bundles a more extensive set of visualization annotations. Other visualization annotations differ from those in Listing 4 in terms of visualization type (e.g., dashb:RealtimeDataVisualization instead of dashb:HistoricalDataVisualization, or the data that the components of the visualizations can accept (e.g. minimum 1 and maximum 10 xsd:double inputs instead of just 1 list of latitude and longitude inputs).

**Listing 4.** Semantic annotation of three visualizations that can be used in the dynamic dashboarding application, serialized in Turtle. The first displays historical positions on a map, the second displays subsequent position observations as routes on a map, and the third displays observations per hour in a table.

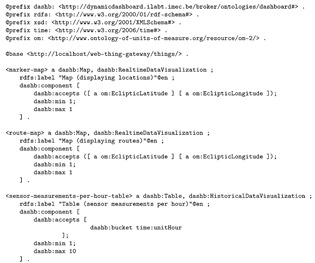



#### 3.4. Annotation of Aggregation Services

Aggregations can calculate statistics, such as a minimum, maximum and average, to summarize large amounts of data. They are particularly interesting in dynamic dashboarding software to display the status of multiple industrial assets across a fleet in one dashboard widget.

Our dynamic dashboarding software also provides web resources for aggregation services and is able to respond upon request with their semantic annotations. Listing 5 bundles the semantic descriptions known for two examples of available aggregations. Note that, again, we use concepts of our custom dashboarding ontology.

**Listing 5.** Semantic annotation of two aggregation services available in the dynamic dashboarding application: one concatenates two sensor observations, the other computes the frequency of a given sensor observation.

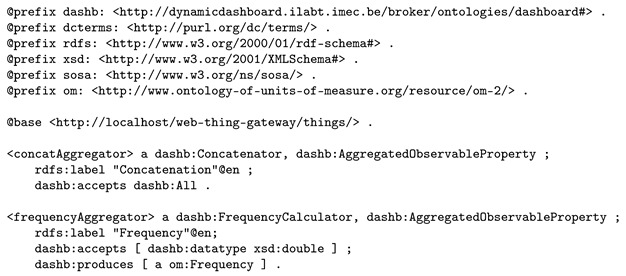



## 4. Matching Sensors and Visualizations

The previous two sections explained that sensors, aggregations and visualizations are all encapsulated as RESTful Web services that can be discovered and that their metadata can be fetched by sending a GET request (with a certain Accept header) to the URL of the service. Using this information, the dynamic dashboarding software collects knowledge about all available Web services, in order to combine them, and, this way, accomplish a visualization task for the user when he/she selects one or more sensors in the graphical interface. For example, one can intuitively see that a thermometer visualization only fits with temperature sensors.

This matchmaking between sensors, visualizations and aggregations is done by combining the gathered service metadata with internally known ontologies, reasoning theorems and logical rules, for example, “a column chart visualizes only quantitative data”. This metadata is expressed in semantic format and passed to a semantic reasoner, which derives new knowledge—the visualizations to suggest. In our application, we use the EYE Reasoner [39,40].

A first input to the reasoning process is the sensor property selection made by the user in the dashboarding user interface. Note that we decided to match available visualizations with a selection of sensor properties, not the sensors, nor the observations they make over time. The reason for that is twofold. The first argument is user-friendliness—a sensor can observe multiple properties, whereas a user may not want to visualize all of them on a single widget, or worse, the visualization may fail to display one or more of the properties linked to a sensor, because it was simply never built for that type of sensor property. The second argument is related to performance. An observed property will make a vast amount of observations over time. Semantifying these and reasoning on them would be nefast for the execution time. As a sensor property usually leads to observations of the same type all the time, and therefore results in the same reasoning conclusions every time, there is no need to execute the reasoning on observation level. In summary, matching visualizations are found when a sensor’s observable property produces a metric with identical characteristics (e.g., data type and metric type) as the one accepted by a visualization component. This required link in the metadata was also indicated in grey on Figure 2.

### 4.1. Use Case—Monitoring the Position of a Train

Section 1 introduced a use case on real-time sensor data provided by a partner in the railway industry. In order to evaluate our research, we simulated the creation of four dashboard widgets that an end user would be interested in, when monitoring the fleet of trains. In these widget creation scenarios, the user respectively visualizes the following in real-time:

1. Wheel acceleration, observed by the SPM sensor on one train
2. Accelerations of a train carriage along three axes, observed by the IMU sensor
3. Average wheel acceleration across three trains, calculated at every incoming timestamp
4. Position of a train

We will demonstrate the semantic reasoning for visualization suggestion on use case 4 below. The complete files involved for this use case, and the others, can be found at http://purl.org/dynamic-dashboard.

The collected data contains latitude and longitude sensor properties, observed by a GPS sensor on the train bogie. Since these are two different properties and the available map visualizations only accept pairs of latitude and longitude observations as input, a concatenation operation must be performed on all latitude and longitude observations to merge them into one single observation. As a result, the observations can be shown on a map of the train’s position or route.

The user’s selection of sensors and aggregation is expressed semantically as in Listing 6.

**Listing 6.** User selection: concatenation of two sensor properties—latitude and longitude.

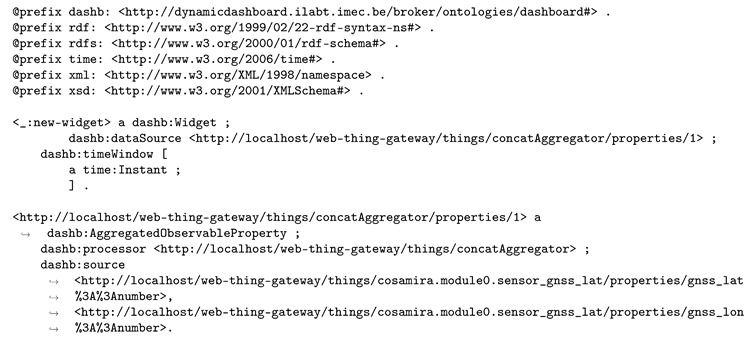



A query file, generated on the fly in this use case, instructs the semantic reasoner about the triples that are expected as result, if they can be derived, see Listing 7. This query file will instruct the reasoner to return the URL and name of visualizations that were found to support the concatenation aggregate as data input and were deemed to be “candidate visualizations”, out of which the user can make a final choice. Note that the serialization format of the query file, and some of the others that will be discussed, is actually N3 [41,42], because of its ability to express logic—for example, inferencing that, if a certain visualizations is proposed as a candidate for the new widget and if that visualization has a name, the implication should be that the name of the visualization is returned, as is the case in Listing 7.

**Listing 7.** The query file instructs the semantic reasoner to return triples containing the URL and name of the visualizations that support the concatenation input and were marked to be displayed as candidate visualizations to the user.

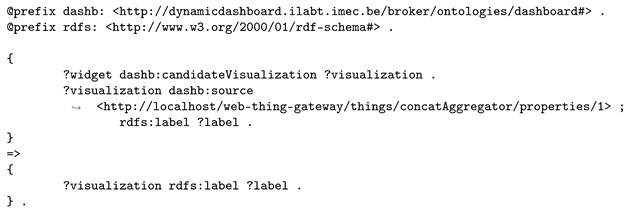



In order for the reasoner to be able to answer the above query, also, the semantic annotation of the concatenation aggregate (see Listing 5) and latitude and longitude sensor properties (see Listing 2) are passed to the reasoner.

Additionally, the metrics taxonomy (see Listing 3 for the relevant part) and annotations of all available visualizations (Listing 4 lists a few) are taken into account.

A reasoning theory about RDFS subclassing is also passed to the semantic reasoner, such that it can derive that instances of specific visualization classes (e.g., dashb:Map) are actually typed with the parent classes (e.g., dashb:Visualization), see Listing 8. We note that this theorem, and many others, are included with the EYE reasoner documentation at http://eulersharp.sourceforge.net/.

**Listing 8.** RDFS subclassing theorem used during the visualization suggestion task.

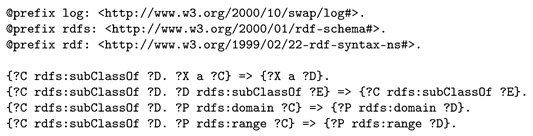



Finally, two sets of logic rules are inputted to the semantic reasoner, one to support aggregations, the other to support visualization. Using all of the above, the reasoner derives new knowledge. Listing 9 presents logic rules that will add support for aggregations as well. The relevant part of the aggregation rules in the train bogie monitoring use case is the following. Since a concatenation service was found, and was presented a list of sources (sensor properties), referring to a metric describing latitude and longitude and their accepted data type, it is concluded that the concatenation service actually produces a list of latitude and longitude metrics.

**Listing 9.** Logic rule involved in the reasoning process to support aggregations on lists of data sources.

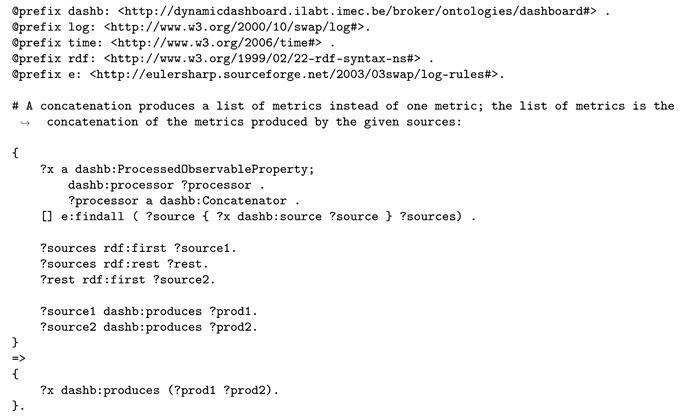



Then, the semantic reasoner uses inference rules for data visualization. In a first step, the reasoner essentially filters the visualizations on their ability to process historical data or real-time data. The widget requirements stated that observations would be provided by the Web Thing per time instant, not in batches (see Listing 6), therefore, the reasoner concludes that the time instants are supported by all RealtimeDataVisualizations, as shown in Listing 10.

**Listing 10.** Logic rule informing the reasoner that only visualizations that display data in real-time can visualize the user’s input.

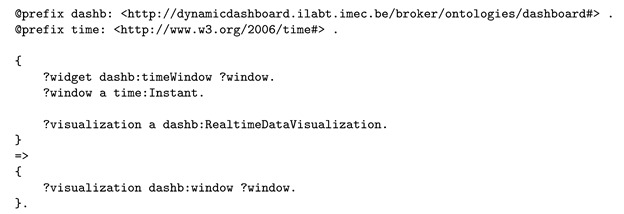



Given that the visualization accepts a list of data inputs and the aggregation service produces a lists of data outputs, a set of rules checks the data inputs in both lists one by one and if they match, the data input is marked as partially supported. If all data inputs were partially supported by the visualization component, the component states that it fully supports the list of data inputs. Listing 11 shows this approach.

**Listing 11.** Inference rules for deciding if a visualization component supports a data source, a concatenation service in this use case, that provides a list of data outputs.

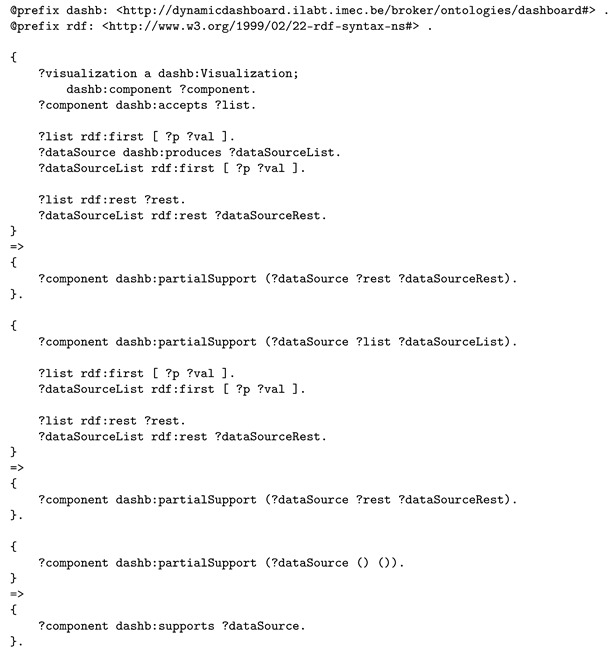



Finally, the semantic reasoner decides that a visualization is an appropriate suggestion if the visualization’s components support a number of data sources (the concatenation in this use case), less than a configured maximum and greater than or equal to a configured minimum amount. The logic rules involved in this can be found in Listing 12.

As a result, the semantic reasoner outputs two map visualizations suggestions—one that can show positions and one that can show routes, see Listing 13. Note that the reasoner was aware of many visualizations (see Listing 4) and will now only suggest two of them to the user, which really aids to prevent creation of nonsensical widgets on a dashboard.

**Listing 12.** Logic rules used for suggesting aggregations.

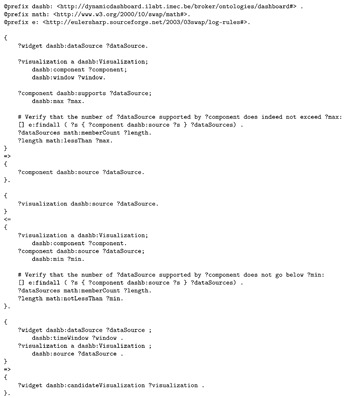



**Listing 13.** Output of the semantic reasoner, stating that two visualizations can display the latitude and longitude sensor input, selected by the user—one that displays subsequent positions as markers on a map, and one that displays routes on a map.

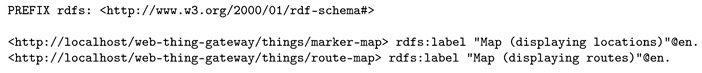



With the deduced visualization suggestions, the user can finish building a new widget for the dashboard. The next section will detail the architecture of our dynamic dashboarding application. After that, we will discuss the results achieved with this application.

## 5. Design of the Dynamic Dashboarding Application

In the previous sections, we presented the discovery of Web Things such as sensors, aggregations and visualizations, using the Web Thing Model. We also demonstrated how these Web Things can be semantically annotated, such that a semantic reasoner can suggest interesting visualizations, when given a selection of sensor(s) and possibly an aggregation service. Here, we will explain how these three drivers are combined into a dynamic dashboarding application.

**Figure 3:**
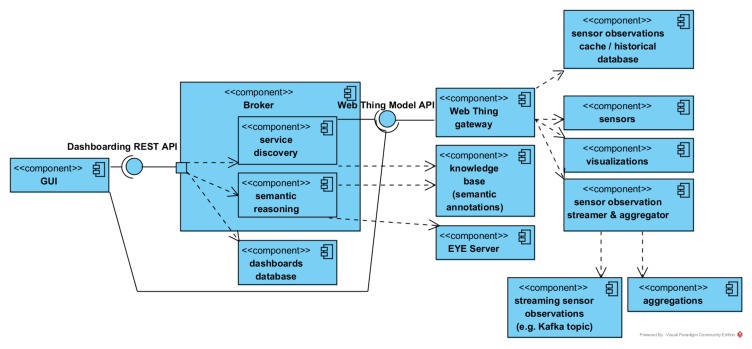
Architecture of the dynamic dashboarding application.

Figure 3 shows the conceptual architecture of our dynamic dashboarding application. It is a more detailed overview than the workflow that was described in the introduction section with Figure 1. The three main promises of the application to accomplish dynamic dashboarding, namely service discovery, semantic annotations and semantic reasoning, are drawn in the center of the figure. These promises will be fulfilled by a collaboration of the user interface (GUI), Broker and Web Thing gateway components. The goal of these main components and their interaction with the components around them will be detailed below.

### 5.1. Web Thing Gateway

A Web Thing gateway is used as a proxy for the available data services (sensors), aggregation services and visualizations for sensor data display, as shown on the right of Figure 3.

The Web Thing gateway essentially offers web resources for each of the available Web Things – sensors, aggregation services and visualizations – through a Web API that is compliant with the Web Thing Model that was discussed in Section 2. This approach makes provided Web Things discoverable, a task that the Broker will manage, with its internal service discovery component.

Our Web Thing gateway implementation supports all three integration patterns (direct, gateway-based and cloud-based connectivity, see Section 2.2) and automatically makes web resources available on the Web Things API, according with the guidelines of the Web Thing Model, for each of the configured Web Things.

A Web Thing gateway also provides, using content negotiation, semantic annotations about the available Web Things on their respective URL. These semantic annotations express the abilities of the provided Web Things, see Section 3.

Because a Web Thing gateway makes sensors, aggregations and visualizations findable and provides the semantic annotations of their abilities in a machine-readable format, the dashboard’s broker component (or any other application that can interpret semantic annotations) can detect the available Web Things and understand how to combine them, for example, setting up a chain of a sensor data source, an aggregation service and finally a visualization.

The end user of the dashboarding application can build dashboards using the knowledge that the Broker gathered during its service discovery. Once the user has added a widget to a dashboard, the dashboard will start communicating directly to the Web Thing gateways involved, such that the chain of required services can be continuously used—first for fetching the latest observation of one or more sensors, then possibly passing the observations to an aggregation service, and loading the visualization into the dashboarding interface and presenting it the processed sensor observations.

Noteworthy to mention is that most of these Web Thing gateways will make use of a cache and/or long-term storage, as drawn in the upper right corner of Figure 3, in order to reduce heavy request traffic to certain Web Things.

The available web resources on a Web Thing gateway can be extended at any moment. The presence and annotations of Web Things must be configured in the Web Thing gateways, or the gateways can discover the available Web Things and their semantic annotations automatically (i.e., decentralized approach), when they reside in the same local network as the gateway (as discussed in Section 2). In an alternative approach, the gateway can just read from a central data publishing repository, for example, a publish/subscribe message bus such as Apache Kafka, which all Web Things write to and read from (i.e., centralized approach).

In the case of fleet monitoring, often many services listen and write data and do this at high frequency. This creates large data volumes at high velocity, two motivators that typically cause scalability problems in big data systems. In such cases, the decentralized approach (service discovery and communication, through a Web Thing gateway) may not meet expectations. The centralized approach however will, using technology that scales, better guarantee a timely delivery of sensor observations to the sensor gateway and thus also in the dashboarding GUI.

For scaling reasons, we used a centralized approach where the aggregation services expect streaming observations on a Kafka message bus, as indicated on in the bottom left corner of Figure 3. Note that only our aggregation services use the centralized approach in our architecture at the moment, since the aggregation operation is costly on large volumes of high-velocity sensor data. Discovery of the aggregation services is done with the Web Thing’s API though.

### 5.2. Broker

The broker is the core of the dynamic dashboarding application. It provides a REST API that covers three functionalities, as indicated on Figure 3.

Firstly, the broker discovers available sensor services, aggregation services and visualization components. The only requirement is that the base URL of a Web Thing gateway is added in the graphical user interface (GUI). The broker then finds the available Web Things by crawling (walking over) all URLs exposed by the Web Thing gateway conform the Web Thing Model, as discussed in Section 2. During this process, the broker gathers the semantic annotations of all found Web Things (as discussed in Section 3) and updates its knowledge base (semantic database, drawn in the center of Figure 3) with it.

Secondly, the broker also reasons, upon request of a user, about what visualizations are interesting matches with the available sensor services. To do so, it reads semantic annotations about the discovered Web Things from its knowledge base and uses, as drawn on Figure 3, EYE Server as the semantic reasoner that must complete the reasoning process, which was described in Section 4.

Finally, through its REST API, the Broker also allows for persisting and serving the state of the dashboards of all users (e.g., created widgets) that users generate when using the dynamic dashboarding application’s user interface. This state is preserved in a database, displayed on the bottom of Figure 3.

### 5.3. Dashboarding GUI

The dashboarding graphical user interface (GUI) component allows a user to build and view a near real-time summary of the operational status of a fleet of appliances or industrial assets. The dashboarding GUI is also called the dashboarding interface; or client, when discussing it in relation to the broker component.

With a pop-up meant for creating widgets, the user can build a dashboard interactively, widget by widget, by selecting sensors and sensor properties to be displayed in the new widget. The dashboarding software will then present visualization options that the broker has reasoned about. Finally, the user can select one of the visualization suggestions and the widget will be added to the dashboard. All data in the pop-up and its drop down menus is automatically filled as a result of crawling and reasoning, so nothing of this GUI is hard-coded nor preconfigured.

During this process, the GUI consecutively makes use of all three functionalities provided by the broker’s API, as described in Section 5.2. Firstly, the GUI retrieves data about the sensors that the broker discovered. Secondly, when the user has selected a sensor and a property observed by the sensor, the GUI fetches several sensor visualization suggestions, made by the broker’s sensor-visualization reasoning component. And lastly, once the user has confirmed all visualization choices for the widget to be created, the parameters are again sent to the broker, which then persists the changes to the dashboard composition in its dashboarding database.

Section 6 will show screenshots of the pop-up that allows a user to create dashboard widgets, and a dashboard that has been constructed with it.

## 6. Results and Discussion—Demonstration and Evaluation of the Dynamic Dashboarding Application

In Section 1, a number of pain points were pointed at in the current state-of-the-art in dynamic dashboarding. As a result, three goals, each with subgoals, were set for this research: (1) building a functional dynamic dashboarding application; (2) reducing human effort with visualization suggestion, using semantic reasoning; and (3) reducing human effort with automated industrial asset management. In what follows, we present and discuss our results for each of these goals.

### 6.1. Goal 1—Build a Functional Dynamic Dashboarding Application

During our research, we built a dynamic dashboarding application for real-time monitoring of industrial fleets. Its architecture was described high-level in Section 1 and in more detail in Section 5.

#### 6.1.1. Goal 1a—Enable Continuous Monitoring of an Industrial Fleet

A first goal was to be able to monitor industrial fleets continuously with the proposed dashboarding platform. So far, we have used it on two projects with industrial partners—HYMOP, bringing together partners interested in fleet monitoring; and DyVerSIFy, comprising of a dynamic dashboarding company, a home ventilation company and a railway technologies company. We selected the train monitoring data of the latter (as described in Section 1) to present and evaluate our dashboarding platform in this paper.

**Figure 4:**
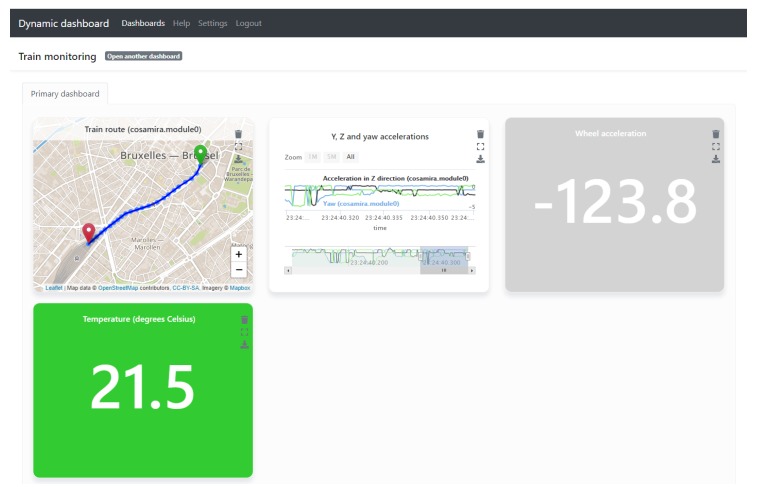
A dashboard constructed for monitoring a train.

Figure 4 shows an example dashboard constructed for this use case. The widgets, from left to right, visualize a train’s position, its acceleration along three axes, its wheel acceleration and the temperature in the train. In the first two widgets, new sensor observations are continuously added, the last two widgets always show the latest sensor observation.

#### 6.1.2. Goal 1b—Enable Interactive Creation of Visualizations

Our dashboard software enables users to create a dashboard by giving it a name and selecting a Web Thing Model-compliant API that is known to the platform. This API is usually served on a gateway that proxies all sensors that monitor an entire fleet of industrial assets, as discussed in Section 2.2. In the platform’s settings page, a user can also register new Web Thing gateways or make the dashboard forget registered gateways.

**Figure 5:**
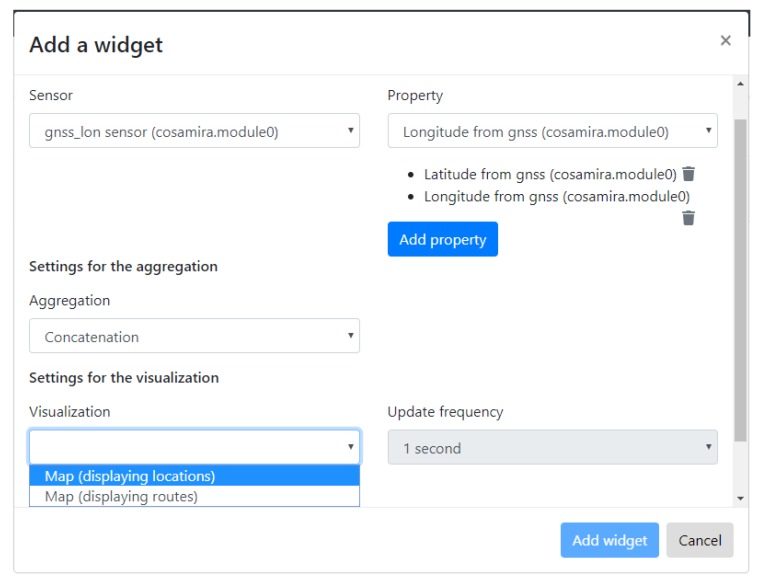
The widget creation pop-up available in the dashboard graphical user interface (GUI), showing the widget configuration the user makes for the use case described in this paper (creating a widget to display the position of a train). The resulting visualization suggestions that are made by the semantic reasoning process are also shown.

Users can interactively build widgets, using a pop-up in the user interface. Figure 5 shows the options that an end user of the platform has chosen in order to create the visualization of the position of a train. The pop-up first fetched all discovered sensors from the Broker’s API and populated the sensors dropdown list with it. When the user selected the latitude sensor from that list, corresponding with a certain train, the properties dropdown list was populated with the properties that the Broker knows are observed by the selected sensor, which is in this case only one latitude property, which is immediately selected. The user repeated this process in order to add the longitude property of the same train to the list of sensor properties to visualize. The pop-up also fetched the discovered aggregation services from the Broker API and the user indicated that a concatenation of the latitude and longitude properties must be visualized. Then, the pop-up contacted requested the Broker API to perform the visualization suggestion using semantic reasoning, as described in Section 4.1 and finally, the two outputted visualization options are displayed in the pop-up. The user can, in this case, choose to visualize the trains positions individually or as a route on a map. The pop-up also allows to select the frequency with which new position observations will be requested (from the sensor’s Web resource) and added to the map visualization.

The result of the user selecting the route visualization in the pop-up, is the first widget on the dashboard that was displayed in Figure 4.

With the pop-up described above, our platform assists the user in interactively composing visualizations. Widgets can also be removed, with the trash bin icon displayed on the top right of every widget (see Figure 4).

A possible extension could be to also suggest aggregations in the widget creation pop-up. The aggregation options to present depend mainly on how many sensors were selected by the user, for example, a multi-sensor average makes no sense when only one sensor property was selected.

### 6.2. Goal 2—Reduce Human Effort with Automated Industrial Asset Management

In this paper, we have explained that industrial fleets evolve fast and that considerable human effort is therefore required to keep a dynamic dashboarding platform aware of changes to the sensors that monitor the fleet. Some dashboards also allow to capture metadata about the assets that sensors monitor. With automated sensor and device discovery, we aim to reduce human effort necessary for these tasks.

#### 6.2.1. Goal 2a—Automate Sensor Discovery

The sensors that a dashboard must monitor may be spread across one or more networks. In Section 2.2, we presented an approach for automatic discovery of services on Internet scale. Sensors are encapsulated as RESTful API resources (Web Things) and a Web API exposes the availability of these sensors and a way for retrieving their observations and metadata using a standardized API contract, the Web Thing Model.

In Section 5, we detailed how an integrator application such as a dynamic dashboard can find out which sensors are available, by crawling the predefined API resources of the Web Thing Model API, once the integrator knows the address of that API. In our approach, the Broker performs the crawling procedure, against Web Thing Model-compliant gateways (Web Thing gateways) that proxy sensors that monitor an industrial fleet.

Many dashboarding platforms allow to add custom data processing services (e.g., aggregations) and visualizations. It is therefore helpful that these, too, can be discovered. Because of that, we proposed to also expose data processing services and visualizations as Web Things through the Web Thing Model API.

Our end goal was to reduce human effort in registering sensor changes in a dashboarding platform, compared to state of the art approaches, in which people must manually register sensor changes in the dashboard interface, or manually register changes in an external fleet management system, after which they are automatically published by some program to an API in the dashboarding platform.

In our approach, end users must only configure the root URL of one or more Web Thing APIs (that proxy sensors, data processing services and visualizations in an industrial fleet) before they can start creating dashboards.

A program that publishes sensor changes to a device management API is no longer required. The registration of Web Thing changes is however shifted to the Web Thing gateway—it must know which Things it must proxy. However, because the Web Thing Model solved the findability problem on Internet scale (the dashboard knows it can communicate to sensors through a Web Thing API), all that remains is the findability of Things on local network scale (the gateway must find the address of the sensor in its network), which is a solved problem. Section 2 listed local network discovery protocols that achieve automatic device (e.g., sensors) detection. This way, the existence of Things must not be registered manually again on the Web Thing gateway level. A drawback of this approach is that a Web Thing gateway must be able to communicate with attached sensors or external services in the right protocols (e.g., REST, MQTT, CoAP, USB, etc.) and must know the data formats involved (e.g., JSON, XML, etc.). Any dashboarding software must do this. But in our approach, this task is now assumed to be implemented in the Web Thing gateway, instead of inside the backend of (or in a plugin for) a chosen dynamic dashboarding platform. Many sensor dashboarding platforms only support a few communication protocols and a lot of companies possess such a wide range of sensor types, that they must often implement plugins for dashboarding platforms anyway. Our approach assumes that this code is integrated in a Web Thing gateway instead.

Another drawback for our automatic discovery approach is that, obviously, the Web Thing gateway must adhere to the Web Thing Model specification. But this specification is rather light-weight.

The amount of sensors monitoring fleets in the industry is often very large and requires enormous effort in managing changes. We think in these cases the advantage of automatic sensor discovery outweighs the extra effort for bundling sensor communication drivers and implementing the Web Thing Model.

#### 6.2.2. Goal 2b—Automate Asset Discovery

Keeping track of changing assets in a fleet, and information about them, also requires a lot of effort. Since our approach comprises semantic sensor metadata annotation on the Web Thing gateway, asset information related to each sensor can easily be added in their annotations, as presented in Section 3.2. The link between sensor and asset is simply represented with a ssn:subSystemOf predicate, and any information about the asset can be added as extra RDF facts.

Because automatic discovery of sensors and Web Thing annotation is part of the solution proposed in this paper, dashboarding platforms can also find asset information linked to sensors and use it to reduce choice overload for the user (e.g., by presenting sensors to select for visualization in a hierarchical overview of the fleet instead of just in a long list), or to improve visualization suggestions (e.g., a thermal image observed in a room may be visualized mapped on the room’s floor plan, to indicate where people are).

### 6.3. Goal 3—Reduce Human Effort with Visualization Suggestion, Using Semantic Reasoning

In Section 1, we explained that commercial dashboarding platforms often offer a plethora of visualizations and have started adopting machine learning techniques to recommend users what the best visualization is for a given sensor selection. We stated that these techniques will not perform well on sensor observations and that, since sensor data must be annotated to be usable for machine learning-based techniques, it can also be semantically annotated in RDF and allow to suggest appropriate visualizations using semantic reasoning instead of machine learning.

#### 6.3.1. Goal 3a—Semantically Annotate Sensors, Data Processing Functionality and Visualizations

In order to be able to suggest visualizations using semantic annotations, we defined the following goals—annotating data types of sensor observations, annotating functionality of visualizations and aggregations and not requiring more effort and technical expertise for metadata annotation.

##### Annotate Data Types of Sensor Observations

In Section 3.2, we demonstrated how we annotate the data types of sensor observations. We explained that it is easier for a dynamic dashboard to match visualizations with the data type of selected sensor properties instead of the data type of all observations involved. Therefore, in sensor property annotations, we state that they produce (dashb:produces predicate) a so-called metric. We make the distinction between quantitative and qualitative metrics and more fine-grained sub types of these, for example, latitude and longitude being a subtype of the quantitative sensor property concept. We introduced a custom taxonomy for metrics that bundles the above concepts and imports the Ontology of Units of Measure (OM-2).

##### Annotate Functionality of Visualizations and Aggregations

In the developed dashboarding ontology, we created a hierarchy of visualization types, see Section 3.3. In the hierarchy, we made the distinction between visualizations that can display real-time data or historical data.

We also added concepts for aggregation functions in our dashboarding ontology, to enable visualization suggestions of aggregated sensor properties. Section 3.4 provided an example annotation of an aggregation.

Further knowledge about the abilities of visualizations and aggregations was added in logic rules (in N3), stating which input data (metrics) they expect and, in case of the aggregations, what the type of output is that they produce, see Section 4.1.

##### Effort and Technical Expertise for Metadata Annotation

With regard to metadata annotation, Section 1 set the objective to not require more effort and technical expertise with the proposed dashboarding solution.

To enable the semantic reasoning in the dynamic dashboard’s Broker component, the Web Thing gateway component must be able to provide semantic annotations about the involved Web Things (sensor properties, visualizations and aggregations) upon request. Human effort is still required to configure these annotations in the Web Thing gateway. Annotation of sensors must also be done in the setup phase of a state of the art dynamic dashboard, albeit in the dashboard’s backend, instead of a Web Thing gateway. Semantic annotation of visualizations and aggregations is new.

In summary, currently, semantic annotation of Web Things will require slightly more effort and technical skill than is the case in commercial dynamic dashboards nowadays. This effort is however comparable to the work that commercial dashboarding platforms have now, while building machine learning-based visualization recommenders (building a supervised data set, trying to obtain good generalization to new visualization queries).

The semantic annotation effort and skill requirements can, however, be greatly reduced. Firstly, an application on the Web Thing gateway can help users to create the semantic annotations using a graphical interface, without knowledge of semantic web technologies. Secondly, more often than not, once a new sensor type is introduced in an industrial sensor network, more sensors of the same type will be deployed later. Therefore, the semantic annotation of these sensors will always be similar and a mapping can be constructed that will generate the sensor annotations for a given sensor type, every time a new sensor of that type is added. Either the annotation mapping can be made by a semantics expert once, before introducing all the sensors of the new type; or the annotation mapping can be "learned by example" from human-made semantic annotations [43] made on one or more already installed sensors.

#### 6.3.2. Goal 3b—Suggest Visualizations for (Aggregated) Sensor Data, Using Semantic Reasoning

One of the main goals in this paper is to suggest visualizations using reasoning on the available semantic annotations of sensor properties, aggregations and visualizations, in order to avoid choice overload (which means less tedious handcrafting of widgets) and creation of nonsensical and non-functional widgets. Also, we wanted to perform the visualization suggestion reasoning within an acceptable time, and we did not want that an end user must have technical expertise to generate and interpret visualization suggestions.

##### Avoid Visualization Choice Overload

When being given the facts about selected sensor properties, the EYE reasoner can deduce visualization suggestions, as illustrated on a demo scenario for aggregated sensor data (a concatenation of latitude and latitude) in Section 4.1. For this scenario, the reasoner outputted that two visualizations are appropriate—one showing the train positions consecutively as dots on a map, and another, specializing in plotting routes.

Knowing that the input data for the visualization was a position, the suggested plots make sense. Also, the visualization options that the user must choose from are reduced from 14 to 2. We note that in a commercial dashboarding platform, more visualizations are available, which will lead to serious reduction of visualization options and therefore elimination of choice overload for the user. Since the visualization options are limited to those that the reasoner deduced to be appropriate for the given input, the dashboarding platform actually assists the user in selecting a visualization that make sense and will function.

We refer to Table 2 for the visualization suggestions on three other scenarios for visualization suggestion on our train monitoring use case. These can be found at http://purl.org/dynamic-dashboard. Scenario 4 is the one that was discussed above. Note that the visualization suggestions in the table again make sense and that the number of options is reduced to respectively 5, 3 and 5 out of 14 for the other three scenarios.

We repeat that, with the semantic reasoning for visualization suggestion, the user is assisted while building dashboards. This makes the process more user-friendly. Also, because each visualization documents its own abilities in its metadata, the complex myriad of if-then-else statements for data visualization in commercial dynamic dashboards is avoided, thereby limiting the software maintenance effort required for improving the dashboard.

##### Acceptable Reasoning Time

Another objective for the proposed dashboard was to keep the required time for reasoning acceptable (<1 s), such that the end user of the dashboard still has a good experience with the platform.

Helping users to prevent them from creating nonsensical dashboard widgets is an interesting feature for a dynamic dashboarding application, however, it is only usable if the delays occurring (while reasoning about the visualization suggestions) are reasonable. Table 2 shows the execution times required for the reasoning alone, the total execution times (i.e., including querying of knowledge bases) and the suggested visualizations, for four use cases. The last use case was described in this paper. The table shows that for all four use cases, the required reasoning times and total visualization suggestion times do not differ substantially across the use cases (the difference in reasoning assignments was not big either) and that all required times (not higher than 800 ms) are still acceptable for an end user of a web application. API response caching can also still be applied in the Broker to avoid calling the reasoner unnecessarily and to thus reduce the required total execution times.

##### Required Technical Expertise

Finally, we set as goal for the proposed dashboard platform that no technical expertise or detailed knowledge (about the fleet, sensors, data processing services and visualizations) should be required.

As Figure 4 and Figure 5 demonstrated, the dashboarding GUI presents the discovered sensors and assists the user in selecting an appropriate visualization. As mentioned in Section 6.2 about human configuration, it is however required that the Web Things are semantically annotated beforehand. This requires knowledge of semantic web technologies (for now, as discussed in Section 6.2) and about the fleet, but is a back-end operation to be performed by experts.

The end user, in summary, must not be a semantics expert or know the fleet really well.

### 6.4. Future Work

In the previous sections, we also discussed some future work directly related to the goals that we set for this research. In this section, we list future work for dynamic dashboarding for industrial fleets in general.

In this paper, we suggested appropriate visualizations using semantic reasoning. The risk of adding widgets to a dashboard that make no sense is now reduced, which saves the user the time of having to reconfigure a new widget. However, the user is still in the loop for creating widgets. The user effort in this user-driven dynamic dashboarding (assisted “handmade” dashboarding) can be further reduced by evolving to automatic dashboard generation. This requires selecting one of the options that were deemed interesting, either at random, or better, the most interesting one, using, for example, recommender systems. Automatic dashboard generation is also particularly interesting when abnormal behavior is detected in industrial systems—experts often only open a dashboard when anomalies occur. If the anomaly alert is coupled with an automatically generated dashboard that presents the interesting KPIs to inspect abnormal behavior (anomaly-driven dashboarding), this improves the user experience a lot. One or more data processing services that detect anomalies in sensor data are part of this solution. We are currently extending our software design to enable anomaly-driven dashboarding.

The architecture presented in this paper can also be extended with other data processing services—for example, conversion, filtering and more aggregation services. We note that currently, aggregation in our architecture (see Figure 3) is only available in a sensor service that accepts streaming sensor data. Part of the solution for supporting more aggregations will be to turn the aggregation into a stand-alone Web API that can accept sensor data from any source—streaming or non-streaming (e.g., retrievable by GET request from a Web Thing Model API). This will require careful engineering, as explained in a note on centralization versus decentralization of services, in Section 5.1.

Also, when extending the types of data processing services (virtual sensors) that can be discovered, the broker will require a considerable evolution in the reasoning process to chain the services together automatically, like pieces of a puzzle, in order to end up with an interesting sensor data visualization. Dynamically finding out which services to combine and in what order, which REST API endpoints to execute and which parameters and parameter values are required could be accomplished by also semantically annotating this extra information in the services, for example, using the FnO ontology [44] and RESTdesc [45,46], or combinations of service description languages and service composition technologies.

Adding dynamic handling of actuators is also an interesting piece of future work, for example, to control ventilation valves, doors, and so forth. remotely from within the dashboard. Particularly interesting for this case is the fact that the dashboard then controls Web Things instead of just listening for their state (bidirectional communication).

Finally, discovery and heterogeneous of protocols and data formats was solved in this paper with the assumption that a Web Thing gateway would proxy the available services and would be Web Thing Model compliant. This means an assumption of a "standard" agreed upon between sensor provider/owner and dashboard provider. If either of these parties does not want to conform with this specific agreement, or if alternative standards should arise, dashboarding software must be able to support many solutions at the same time, or find other ways for discovering sensors over the Internet and finding out automatically how to handle heterogeneous protocols and formats.

## 7. Conclusions

Dashboards present and communicate the condition of monitored environments to supervising experts. In the case of monitored industrial fleets, the often vast and rapidly evolving fleet motivates the choice for dynamic dashboards—they allow users to freely and interactively create visualizations. The proposed platform enables dynamic data visualization. State-of-the-art dynamic dashboarding platforms however require a lot of human effort to manage changes to a fleet and a plethora of available visualization types may lead to choice overload for the user or to the creation of nonsensical or non-functional visualizations.

In this paper, we presented a dynamic dashboarding platform for continuous monitoring of industrial fleets that tackled both aforementioned problems, by adopting Semantic Web of Things technologies.

Sensors, linked assets, data processing services such as aggregations and visualizations are discovered automatically, because they are encapsulated as web resources (Web Things), exposed through a REST API that is compliant with the Web Thing Model. Because of the imposed standard API resources, the dynamic dashboard can crawl the API to discover the resources it exposes, once a user registers the address of he API’s root resource in the dashboard’s user interface. Manually registering sensor changes in the dashboard or another system (which publishes changes to a sensor management API) is no longer required. However, sensor protocol and data format drivers and the Web Thing Model specifications must be implemented in the Web Thing API, but we argued that the extra work nicely trades off with the elimination of continuously registering sensor changes manually for large industrial fleets.

Also, semantic annotations are provided on the Web Thing API about the data types that sensor properties produce and the data types that aggregations and visualizations support. Annotations were made with a data types taxonomy, the OM-2 ontology, a dashboarding ontology and N3 rules. Semantic reasoning about these annotations allows the platform to suggest appropriate visualizations for a selection of sensor properties and aggregations. As a result, the large set of visualization options is reduced and construction of nonsensical dashboard widgets is avoided. In contrast with current commercial dashboarding platforms, visualizations and aggregations must also be annotated, which requires slightly more human effort and technical skill. We however suggested two ideas to overcome this—a user interface that allows users to interactively create semantic annotations without knowing semantic technologies, and annotation by example (annotating new sensors automatically when similar sensors with annotations already exist). We demonstrated that the suggested visualizations make sense and reduce the tediousness in handcrafting dashboard widgets, that reasoning times were below 1 second and that the end user of the dashboarding interface does not need to have technical expertise or detailed knowledge about the sensor data.

All files involved in the semantic reasoning for visualization suggestion are available online at http://purl.org/dynamic-dashboard, including the dashboarding ontology.

We used the proposed dynamic dashboarding platform on two projects with industrial partners and showcased it on a train monitoring use case in this paper.

To the best of our knowledge, we are the first to present a dynamic dashboarding platform that combines automated Web Thing discovery and semantic reasoning for visualization suggestion. Such a dashboarding platform proves to be a very powerful monitoring tool for complex, hard to access and/or critical environments.

## Figures and Tables

**Table 1:** The Web API resources that a RESTful sensor API must provide, in order for it to be compliant with the Web Thing Model contract.

URL	Goal
{wt}	The root resource URL of a Web Thing.
{wt}/model	The model of a Web Thing, listing of properties, actions and things.
{wt}/properties	The list of properties observed by the Web Thing. Most probably provided when the RESTful API for the Web Thing is hosted by the Web Thing itself (direct connectivity integration pattern).
{wt}/properties/{propertyId}	A specific property
{wt}/actions	The list of actions, in case the Web Thing can control actuators, e.g., can open or close an exhaust.
{wt}/actions/{actionId}	A specific action
{wt}/actions/{actionId}/{executionId}	A specific execution of an action
{wt}/things	Lists other Web Things that the Web Thing proxies for, most probably when the Web Thing is set up conform the gateway- or cloud-based connectivity patterns.
{wt}/things/{thingId}	A specific Web Thing proxied by the current Web Thing.
{wt}/subscriptions	List of subscriptions to actions or properties, by clients that want to be informed when the state of this action or property changes.
{wt}/type	URL for information about the type of Web Thing
{wt}/product	URL for product info about the Web Thing
{wt}/help	Resource for a help page about this Web Thing
{wt}/ui	Resource for a HTML-based user interface for this Web Thing
{wt}/...	custom resource

**Table 2:** Execution times required for the semantic reasoning process to complete the suggestion of visualizations, given a certain input of sensor properties and, possibly, an aggregation. The last column displays which visualizations were recommended by the reasoner.

Case	Sensor Properties	Aggregation	Reasoning Time (ms) (mean ± SD, 50 runs)	Total Time (ms) (mean ± SD, 50 runs)	Suggested Visualizations
1	wheel acceleration of one train	no aggregations	585.4 ± 61.3	695.6 ± 61.3	Column chart Gauge (modern style) Gauge (traditional style) Line chart (updating at observation frequency) Textual display of observations Smiley visualization
2	accelerations of a train bogie along three axes	no aggregation	578.4 ± 26.6	685.3 ± 29.5	Column chart Line chart (updating at observation frequency)
3	wheel accelerations of three train bogies	multi-sensor average	582.7 ± 33.2	694.3 ± 35.7	Column chart Gauge (modern style) Gauge (Traditional style) Line chart (updating at observation frequency) Textual display of observations Smiley visualization
4	latitude and longitude of a train bogie	concatenation	582.4 ± 35.3	693.8 ± 36.2	Map (displaying locations) Map (displaying routes)

## Abbreviations

The following abbreviations are used in this manuscript:

API	Application Programming Interface
CSS	Cascading Style Sheets
CoAP	Constrained Application Protocol
DHCP	Dynamic Host Configuration Protocol
DLNA	Digital Living Network Alliance
DNS	Domain Name System
DNS-SD	DNS Service Discovery
EYE	Euler Yet another proof Engine
FaBiO	FRBR-aligned Bibliographic Ontology
GPS	Global Positioning System
GUI	Graphical User Interface
HATEOAS	Hypermedia as the Engine of Application State
HTML	HyperText Markup Language
HTTP	Hyptertext Transfer Protocol
IP	Internet Protocol
IoT	Internet of Things
JSON	JavaScript Object Notation
JSON-WSP	JavaScript Object Notation Web-Service Protocol
KPI	Key Performance Indicator
mDNS	multicast DNS
ML	machine learning
MQTT	MQ Telemetry Transport
OM-2	Ontology of units of Measure, version 2
OP	Observable Property ontology
OWL	Web Ontology Language
OWL-DL	OWL Description Logic
QUDT	Quantities, Units, Dimensions and Types ontology
RDF	Resource Description Framework
RDFS	RDF Schema
REST	Representational State Transfer
SIO	Semanticscience integrated ontology
SOSA	Sensor, Observation, Sample, and Actuator ontology
SPARQL	SPARQL Protocol And RDF Query Language
SSN	Semantic Sensor Network ontology
SWRL	Semantic Web Rule Language
SWoT	Semantic Web of Things
TCP	Transmission Control Protocol
UPnP	Universal Plug and Play
URL	Uniform Resource Locator
W3C	World Wide Web Consortium
WSDL	Web Services Description Language
WoT	Web of Things
XML	Extensible Markup Language
